# Expression and prognosis of ADAMTS18 in different tumors

**DOI:** 10.3389/fonc.2024.1347633

**Published:** 2024-02-28

**Authors:** Wenfei Guo, Yuying Zhang

**Affiliations:** School of Biological Science and Technology, University of Jinan, Jinan, China

**Keywords:** ADAMTS18, tumor microenvironment, prognosis, invasion, migration

## Abstract

ADAMTS18 has been identified as an orphan member of the ADAMTS (a disintegrin and metalloproteinase with thrombospondin motifs) family of Zn-dependent secreted metalloproteinases since 2002. Despite the recent breakthroughs in tumor biology of ADAMTS18, there is no literature systematically discussing the relationship between ADAMTS18 and cancer. In this review, we will summarize the expression pattern and prognostic value of ADAMTS18 in various cancers. In addition, we will highlight the biological functions of ADAMTS18 in the tumor microenvironment, including the regulation of cell proliferation signals, death patterns, invasion, and migration, which influence cancer progression.

## 1 Introduction

ADAMTS proteases consist of 19 secreted metalloproteinases that have been implicated in oncogenic and tumor-protective functions (1–3). In recent years, there has been an increase in the number of known substrates of ADAMTS family members secreted by cancer and stromal cells (4–12). A variety of biological functions such as cell proliferation, migration, invasion, and angiogenesis have been attributed to the interaction of these enzymes with regulatory factors or to the cleavage of extracellular matrix components through their protein hydrolase activity (7, 13–18).

Initially, the link between ADAMTS18 and cancer was based on the altered mode of action of ADAMTS18 in different types of cancer (19–21). However, new evidence suggests that the complexity of ADAMTS18’s mode of action in cancer has extended to fine-tuned factors in cell signaling pathways and tumor microenvironment. Here, we will focus on the latest research advances to systematically introduce the biological functions and mechanisms of ADAMTS18 in tumorigenesis and development, as well as its close connection with tumor diagnosis and prognosis. It is hypothesized that fibronectin, as a candidate substrate and interacting protein, may be involved in the regulation of the tumor microenvironment by ADAMTS18 (22, 23). To date, there is a gap in clinical targeted drug studies for ADAMTS18, and we will provide examples of ADAMTS18 in combination with cisplatin and curcumin to provide new references for tumor therapy (24–28).

## 2 The domain organization of ADAMTS18

**Figure 1:**
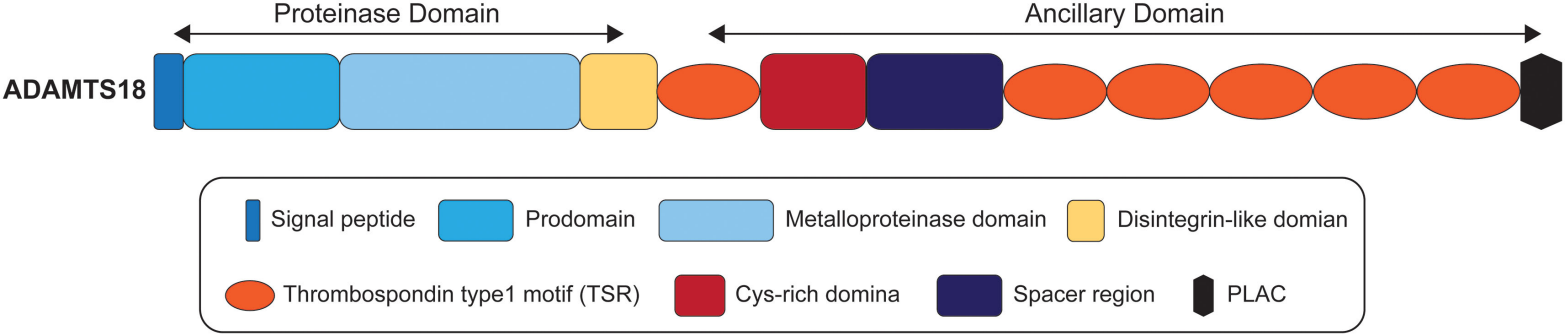
The domain organization of ADAMTS18.

ADAMTS proteases share a multi-domain organization that includes a signal peptide, a prodomain, a metalloproteinase domain, a disintegrin-like domain, a thrombospondin type1 motif (TSR), a Cys-rich domain, and a spacer region (1, 29). It has been reported that the ancillary domains of some ADAMTS proteases mediate substrate recognition (30–33), but it is not clear whether ADAMTS18 is consistent with this phenomenon. As illustrated in **Figure 1**, the ancillary domain of ADAMTS18 is composed of the first TSR, Cys-rich domain, spacer region, five additional C-terminal TSR repeats, and protease and lacunin (PLAC) motif (34). All ADAMTS proteases have a furin cleavage site which releases mature proteins by cleaving prodomain, and ADAMTS18 is no exception (35). In addition, there is a thrombin cleavage site between Arg775 and Ser776 in the spacer region of ADAMTS18, which releases the C-terminal 45-kDa platelet active fragment composed of five TSR repeats and PLAC motif after being cleaved by thrombin (35, 36). Biochemical and 3-dimensional structural data showed that the members of the ADAMTS family have a catalytic mechanism similar to that of MMP and ADAM, involving three conservative His residues and zinc atoms (37, 38). In addition, a unique property of ADAMTS proteases is found in the crystal structures of the catalytic domain of ADAMTS4 and 5, which can balance the open structure that binds Ca^2+^ and the closed structure that releases Ca^2+^ (2). In these two conformations, ADAMTS proteases may regulate their catalytic actions via binding accessory proteins and substrates (2).

## 3 Expression and prognosis of ADAMTS18 in different tumors

Data from Gene Expression Profiling Interactive Analysis (GEPIA) suggest that ADAMTS18 is associated with cancer because of their altered expression in different tumors. ADAMTS18 is located at 16q23.1 in the human genome, and heterozygous deletions in the 16q23 region have been strongly associated with a variety of cancers (19, 39, 40). Increasing evidence suggests that hypermethylation of the ADAMTS18 promoter CpG Islands (CGI) leads to epigenetic inactivation in a variety of cancers, thus making ADAMTS18 a tumor suppressor gene (TSG) (19, 41). ADAMTS18 has been reported to have tumor suppressor activity in esophageal adenocarcinoma, nasopharyngeal carcinoma, colorectal carcinoma, breast carcinoma, lung carcinoma, cervical carcinoma, and clear cell renal cell carcinoma (19, 28, 42–45). Furthermore, the methylation frequency of ADAMTS18 varies in a variety of tumor tissues (40, 43). For example, the methylation frequencies of ADAMTS18 in breast, colorectal, and pancreatic cancers are 70.8%, 49%, and 39%, respectively, which may explain the mechanism of action of ADAMTS18 in different types of cancers (40, 43). In addition to promoter methylation, the gene mutation is another way to inactivate ADAMTS18. A comprehensive mutation study identified two missense mutations (R382K and K455T, both located in the catalytic region of metalloproteinases) in ADAMTS18 in colon cancer (46, 47). Moreover, in human melanoma, 19 members belonging to the ADAMTS family were sequenced, with ADAMTS18 having the highest mutation frequency (20). Notably, ADAMTS18 was found to be upregulated in gastric adenocarcinoma and pancreas adenocarcinoma tissues, suggesting that ADAMTS18 also has oncogenic activity (21, 48).

Cancer recurrence and metastasis is a major problem for cancer patients. Therefore, it is significant to explore independent prognostic indicators for cancer intervention and treatment. In the large number of primary cancer samples, ADAMTS18 downregulation was found to be the result of promoter methylation, and gene methylation is an ideal biomarker for early diagnosis or efficacy monitoring (19, 49–52). Since these changes occur in the early stages of cancer, it has been hypothesized that ADAMTS18 can aid in early cancer diagnosis and prognosis. For example, high expression of ADAMTS18 in lung cancer is positively correlated with high overall survival (OS) in -stage patients (28). In addition, low expression of ADAMTS18 in cervical cancer is positively associated with high tumor stage, positive lymph node metastasis, distant metastasis, short OS, and disease-free survival (DFS) (44). In gastric adenocarcinoma, ADAMTS18 is positively correlated with tumor differentiation, lymph node metastasis, TNM stage, short OS (21, 53). The immune positivity of ADAMTS18 was also found to be higher in metastatic lymph nodes than in non-metastatic lymph tissue in pancreas adenocarcinoma (48). In addition, ADAMTS18 in invasive ductal carcinoma (IDC) of the breast is correlated not only with tumor histological grade but also with estrogen receptor (ER), progesterone receptor (PR), and Ki67, which are markers of breast cancer (54). If ER and PR are positive in tumor tissues, their proliferation is hormone-dependent, and high expression of ER and PR is associated with a better prognosis (55). According to a hypoxia risk model which has been proposed for the diagnosis and prognosis of ESCC, we can predict 1-, 3-, and 5-year survival of patients, and ADAMTS18 is one of the key hypoxia-related genes (HRGs) of this model (56). In conclusion, ADAMTS18 may be a good prognostic factor for many cancer types (**Table 1**).

**Table 1:** The prognostic value and function of ADAMTS18 in different tumors.

Type of cancer	Role	Prognostic value	Functions	Ref.
Lung cancer	Tumor-suppressing	High OS T1 patients	Inhibit cell proliferation, migration, and invasion; promote cell apoptosis, and increase the cisplatin sensitivity of cells	(28)
Cervical cancer	Tumor-suppressing	Negatively correlated high tumor stage, positive lymph node metastasis, distant metastasis, short OS, and DFS	Suppress cervical cancer progression and metastasis	(44)
Melanoma	Tumor-suppressing		Mutant ADAMTS18 promotes growth factor-independent cell proliferation, increases cell migration, and increases metastases *in vivo*	(20)
Colitis-associated colorectal cancer	Tumor-suppressing		ADAMTS18 deficiency promotes cell proliferation and inhibits cell apoptosis, enhances tumorigenesis and intestinal inflammation	(42)
Breast cancer	Tumor-suppressing	ER, PR, and Ki67 and a severe histological grade	Inhibit cell migration and invasion, inhibit metastases *in vivo*	(43), (54), (57)
Renal clear cell carcinoma	Tumor-suppressing		Induce ferroptosis/apoptosis, inhibit cell proliferation, and migration, increase the sunitinib/axitinib sensitivity of cells, inhibit immune escape	(24–27), (45)
Esophageal carcinoma	Tumor-suppressing	A critical HRG of a hypoxia risk mode	Inhibit cell proliferation	(19), (56)
Gastric adenocarcinoma	Tumor-promoting	Tumor differentiation, lymph node metastasis, and TNM stage and negatively associated with OS		(21), (40), (53)
Pancreatic cancers	Tumor- promoting	A higher expression in metastatic lymph node tissue		(48)
Nasopharyngeal carcinoma	Tumor-suppressing		Inhibit cell proliferation	(19)

## 4 Effects of ADAMTS18 in tumor

ADAMTS18 has been widely documented to play a role as a TSG in most types of cancer. In this context, the large number of tumor cells and mouse tumor models have been established and analyzed for the function of ADAMTS18, including cell proliferation signals, cell death patterns, cell migration, and invasion. In the AOM/DSS-induced colitis-associated colon cancer (CAC) mouse model, ADAMTS18 gene deletion promotes cancer cell proliferation and inhibits cancer cell apoptosis (42). An ADAMTS18 mutant melanoma cell line was established by simulating standard conditions *in vivo* (20). In addition, mutant melanoma cells reduce their dependence on factors required for cell growth, suggesting a growth-promoting effect of mutant ADAMTS18 on melanoma (20). Mutant ADAMTS18 also promotes migration and invasion of melanoma cells *in vitro* by reducing adhesion to laminin-I (20). This was further confirmed by histopathological results of subcutaneous injection tests in nude mice (20). In the sunitinib-resistant clear cell renal cell carcinoma (ccRCC) cell model, ADAMTS18 was shown to down-regulate the expression levels of NCOA4, FTH1, and p53, and induced ferritin deposition to inhibit proliferation (26). In most cases, the tumor suppressor protein p53 (TP53) inhibits iron oxidation through multiple pathways (58). NCOA4 and FTH1 mediate ferritin phagocytosis, and decreased levels of their expression increase ferritin levels and induce ferroptosis (59, 60). In addition, similar results were observed in axitinib-resistant cells and animal models, where ADAMTS18 inhibited the development of ccRCC (27). In renal clear cell carcinoma, there is positive feedback regulation between ADAMTS18 and miR-148 (25). Therefore, ADAMTS18 could play a tumor-suppressive role in renal clear cell carcinoma by inhibiting autophagy (61). These are summarized in **Table 1**.

## 5 The mechanisms of ADAMTS18 in tumor

**Figure 2:**
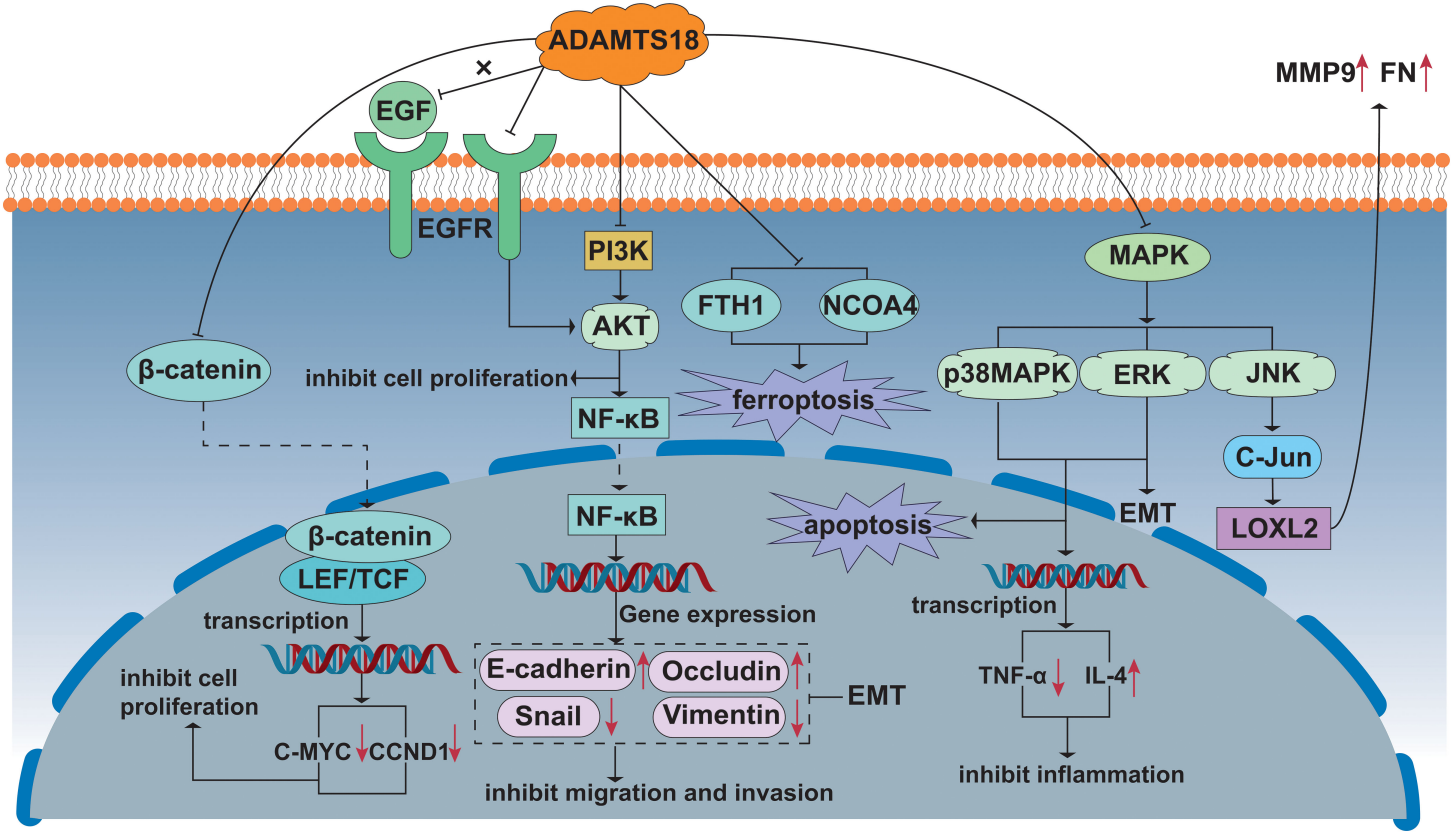
Signal pathway including ADAMTS18 related to cancer. Wnt/β-catenin signal pathway: ADAMTS18 prevents the transfer of β-catenin from the cytoplasm to the nucleus and binds to transcription factors, hindering the transcription of downstream target genes CCND1 and C-MYC, and the growth of cancer cells is inhibited. PI3K/AKT/NF-κB signal pathway: ADAMTS18 inhibits the PI3K/AKT/NF-κB signal pathway, inhibiting cancer cell migration and invasion by EMT. On the other hand, ADAMTS18, as an antagonist of EGFR, also inhibits AKT signal transduction. MAPK signal pathway: ADAMTS18 promotes apoptosis, inhibits inflammation, and exhibits anti-tumorigenic by inhibiting the ERK/P38 MAPK signal pathway. ADAMTS18 is shown to down-regulate the expression of NCOA4 and FTH1 protein levels and induce ferroptosis in cancer cells. In addition, the ERK signal pathway also promotes EMT. ADAMTS18 inhibits cell secretion of MMP9 and FN by inhibiting JNK signal pathway activity and reducing LOXL2 protein level.

In recent decades, previous studies have made breakthroughs in the tumor suppressor activity and mechanisms of ADAMTS18. Multiple intracellular signaling pathways, such as NF-κB, AKT, EMT, Wnt/β-catenin, p38 MAPK/ERK1/2, and epidermal growth factor receptor (EGFR) assist ADAMTS18 to play important roles in different types of tumors (28, 42, 43). *In vivo* and *in vitro* experiments have shown that ADAMTS18 inhibits migration and invasion of breast cancer cells (43). In this process, breast cancer cells were found to exhibit an epithelial phenotype, with higher expression of the epithelial marker E-cadherin and down-regulated expression of the mesenchymal marker Snail (43). The AKT kinase and NF-κB signaling pathways make it one of the most common pathways in cancer. In addition, AKT-dependent activation of the NF-κB signaling pathway has been shown to induce EMT (62). Following previous studies, ADAMTS18 showed tumor suppressive effects by inhibiting epithelial-mesenchymal transition through inhibiting the NF-κB/AKT signaling pathway in breast cancer (43). In the HER2 transgenic spontaneous mammary tumor mouse model, we found a similar conclusion that ADAMTS18 deletion leads to the enhancement of integrin-mediated PI3K/AKT, ERK, and JNK signal activity, which increases the risk of mammary hyperplasia and breast cancer occurrence and metastasis (57). In the AOM/DSS-induced CAC mouse model, the number of β-catenin, cyclin D1, and c-myc positive cells is increased in ADAMTS18 KO mice compared to WT littermates, whereas the expression of E-cadherin is decreased (42). The Wnt/β-catenin signaling pathway plays an important role in the development, progression, metastasis, and invasion of CRC (63). When the amount of β-catenin in the cytoplasm steadily accumulates, β-catenin is transferred from the cytoplasm to the nucleus and binds to transcription factors, initiating the transcription of downstream target genes cyclin D1 and c-myc (64). E-cadherin is known to be an epithelial marker, downregulation of E-cadherin causes epithelial cells to lose polarity and adhesion, promoting tumor migration and invasion (65, 66). In addition, in CAC, E-cadherin binds to β-catenin to form a complex that binds to the cytoskeleton and prevents β-catenin from translocating to the nucleus, thus preventing the activation of the Wnt/β-catenin signaling pathway (67). Histological results from the CAC model showed that ADAMTS18 deletion up-regulates phosphorylated p38 MAPK and ERK1/2 expression (42). The tumor-suppressive mechanism of some ADAMTS family members, such as ADAMTS8 and ADAMTS12, is manifested by antagonizing ERK signaling (68, 69), and the ERK/P38 MAPK signaling pathway inhibits tumor growth in colon adenocarcinoma (70). In addition, the function of ERK/P38 MAPK in regulating cell proliferation and apoptosis has been reported (71). Down-regulation of ADAMTS18 promotes apoptosis and promotes colorectal cancer cell proliferation, which is consistent with the above phenomenon (42). Taken together, the down-regulation of ADAMTS18 promotes colon cancer development and progression by promoting Wnt/β-catenin and p38 MAPK/ERK1/2 signaling pathways (42). Furthermore, the mechanism by which ADAMTS18 increases the sensitivity of lung cancer cells to cisplatin is that ADAMTS18 acts as an inhibitor of epidermal growth factor receptor/protein kinase B (EGFR/AKT) signaling antagonist (28). In conclusion, understanding the molecular mechanism of ADAMTS18 as a TSG will help to develop new therapies targeting tumorigenesis and metastasis. These are also described in **Figure 2**.

## 6 ADAMTS18 and tumor microenvironment

The tumor microenvironment places cancer cells within a network of stromal cells that are comprised of fibroblasts vascular cells and inflammatory immune cells (29, 72, 73). In addition, extracellular matrix (ECM) and cytokines/growth factors are representative components of TME (29). TME regulates tumor proliferation and invasion, angiogenesis, inflammation, immune escape, and drug resistance through the modulation of this complex system (74). ADAMTS is an enzyme that degrades stroma. Tumor cells degrade stroma by secreting ADAMTS, which in turn promotes tumor cell invasion and metastasis. In addition, inflammatory cells and fibroblasts in the tumor microenvironment can also secrete ADAMTS, which plays an important role in promoting tumor development. Secreted or membrane-associated metalloprotease activities have been traditionally associated with an increase in the tumorigenic potential of tumor cells (75). These ADAMTS can modify the primary tumor microenvironment by proteolytic-dependent or independent mechanisms. About ADAMTS18, research in this area is still vague. Thus, only four aspects of inflammation, immunity, microvessel formation, and extracellular matrix are described next.

### 6.1 Carcinogenic inflammation

When cancer cells as well as surrounding stromal cells interact with inflammatory cells, an inflammatory tumor microenvironment (TME) is formed (76). Chronic inflammation has been characterized as one of the markers of cancer, inflammation contributes to various stages of tumor formation (77, 78). Recent studies have shown that pro-inflammatory factors and pro-inflammatory mediators produced by tumor cells maintain tumor cell proliferation and survival as well as immune escape (79, 80). Under different non-oncological conditions, ADAMTS protease is directly involved in inflammatory response in multiple ways (81). In some previously published data, ADAMTS18 KO mice exhibited a higher degree of inflammatory infiltration, increased expression of the inflammatory factor TNF-α, and down-regulate expression of the anti-inflammatory factor IL-4 in a DSS-induced colitis model compared to wild-type mice (42). Thus, disruption of ADAMTS18 signaling promotes intestinal inflammation in mice, which in part creates a tumor-promoting microenvironment for mice.

### 6.2 Immune infiltration

Few studies have illustrated that ADAMTS proteases are directly involved in tumor immune response, but they play a regulatory role in the infiltration and polarization of specific immune cell groups (81). Tumor ECM promotes immunosuppressive activity and maintains immune escape mechanisms of cancer (81). To our knowledge, most substrates of ADAMTS protease are ECM. It is important to note that many substrates of ADAMTS protease have been proven to be directly and indirectly involved in the research of the immune system (81). There are two typical representatives of immune cells in the tumor microenvironment: antitumor cells and tumor-promoting cells (82, 83). It is well known that effector T cells (including cytotoxic CD8+ T cells and effector CD4+ T cells), natural killer cells (NK), dendritic cells (DCs), and M1-polarized macrophages have anti-tumor effects (84). However, tumor-promoting immune cells such as Tregs, MDSCs, M2-polarized macrophages, N2-polarized neutrophils, type 2 natural killer T cells (NKT2), and ILC2 are also beginning to come into the limelight (73, 84). In some cases, tumor progression depends on the balance between these two types of typical immune cells. Studies on immunization with gastric adenocarcinoma gene vaccines have shown that upregulation of ADAMTS18 enhances the infiltration of CD8+ T cells, CD4+, macrophages, and neutrophils, which induces an immune response and positively correlates with the immune infiltration of DCs (85). As the most potent specific antigen-presenting cells (APCs), dendritic cells initiate adaptive immune responses and antitumor responses by activating T cells (85). PD-1 receptors are mainly expressed on the surface of activated T cells. Normally, they can inhibit T cell activity by binding to PD-L1 or PD-L2 ligands on the surface of normal cells, preventing unintended damage to normal cells (86). However, tumor cells can evade immune response by bypassing the immune surveillance of T cells through upregulation of PD-L1 (83, 87). In a study of axitinib-resistant ccRCC mice, ADAMTS18 may act as a similar immunosuppressor by reducing immune escape and enhancing anti-tumor immunity through the up-regulation of the ratio of CD8+ and CD4+ T cells as well as the down-regulation of the ratio of CD45+/PD-L1 (27, 88).

### 6.3 Tumor angiogenesis

The infinite passage and rapid proliferation of tumor cells require blood to transport a large amount of nutrition and oxygen, so tumor growth depends on the rapid formation of tumor blood vessels (89, 90). There were some reports concerning the role of ADAMTS family members in angiogenesis. ADAMTS2, 5, 8, 9, 12 have been demonstrated to be involved in inhibiting angiogenesis and/or cancer, as well as ADAMTS1,4,13 exhibit both pro-angiogenic and anti-angiogenic activity (35, 75). At the same time, ADAMTS18 has been proven to be an important participant by affecting angiogenesis. In ADAMTS18-deficient zebrafish and mouse models, ADAMTS18 is associated with defective angiogenesis and vascular malformations (91–93). The detrimental factors resulting from ADAMTS18 deficiency include the loosening of adhesion between the endothelial cells and the vascular basement membrane, which accelerates FeCl3-induced carotid thrombosis and exacerbates cerebral infarction following ischemia in mice (91). ADAMS18 deficiency leads to activation of the Notch3 signaling pathway and promotes differentiation of cranial neural crest cells (CNCCs) to vascular smooth muscle cells which contribute to angiogenesis (93). Decreased vascular integrity and functional defects eventually lead to hypoxia and acidification of the tumor microenvironment (TME), which increases tumor spread and metastasis (94). In addition, ADAMTS18 affects vascular phenotype by regulating Sit/Robo, DLL4/Notch, COX2, and FGFR signaling (92). Slit/Robo family and COX2 molecules have been proven to be involved in tumor angiogenesis and promote tumor progression. DLL4/Notch is an indispensable pathway in the early stage of pathological and physiological angiogenesis. FGFR mediates angiogenesis of target cells by autophosphorylation and activation of downstream Src family kinases. The latest studies have identified ADAMTS18 as a target gene of the endothelial-specific super-enhancer SE12313 (95). The down-regulation of ADAMTS18 has been shown to inhibit angiogenesis, which is reflected in a decrease in endothelial cell sprouting (95). These results establish a link between ADAMTS18 and angiogenesis.

### 6.4 Extracellular matrix

The ECM is a complex meshwork of collagen fibers, elastin fibers, fibronectin, laminin, proteoglycans, glycoproteins, and other matrix proteins (96). ECM remodeling regulated by tumor cells and other stromal cells plays an indispensable role in tumor spreading and metastasis (97, 98), and this process is often accompanied by changes in ECM mechanobiological signals (99). Elastic fibers, collagen fibers, and glycosaminoglycans (GAGs) are the three main components that determine the mechanical composition of the ECM, with collagen fibers giving stiffness and strength to the connective tissue, and elastin fibers giving ductility and elasticity to the tissue (100). According to recent insights, ADAMTS18 has tremendous biological effects on the ECM itself and its mechanical stimulation. An important research advance has shown that ADAMTS18 gene deletion up-regulates the levels of collagen I, collagen IV, laminin, and fibronectin. Fibronectin is the target for specific cleavage by the ADAMTS18 enzyme, in breast tissue (22). Fibronectin not only guides the assembly of collagen I, collagen IV, and laminin (101, 102) but also plays a major structural role in the binding of fibronectin to microfibrils (103). Thus, the absence of the metalloproteinase ADAMTS18 promotes the accumulation of fibronectin, leading to the deposition of ECM proteins, including collagen I and IV, which is associated with ECM stiffness (22). In the HER2 transgenic spontaneous mammary tumor mouse model, ADAMTS18 deficiency causes the deposition of mammary ECM molecules, including laminin (LN-511), FN, and type I collagen (57). FN can affect EMT through the ERK pathway (104). In addition, the deposition of FN in the basement membrane is related to the lymphatic metastasis of breast cancer (105). LN-511 is thought to be responsible for tumor migration and invasion through integrin receptor-mediated signaling pathways (106). As a consequence, the relationship between extracellular matrix changes and tumor behavior has been established. ADAMTS18-based ECM stiffness activates mechanoreceptors represented by the transmembrane receptor integrin (107) and affects the assembly of cytoplasmic complexes composed of focal adhesion kinase (FAK) and the scaffolding protein actin (108). With the assistance of ADAMTS18, the relationship between mechano-signaling and cytoskeletal assembly is established to regulate the malignant phenotype of tumor cells. Further studies demonstrated that ADAMTS18 binds to fibrillin-1, the major protein of microfibrils, affecting the assembly of F-actin and ultimately altering the migratory capacity of cells (23). It has been determined that ADAMTS18 directly affects the TME through its substrate fibronectin or its interacting protein fibrillin-1 (22, 23). Yet it’s worth noting that the protease activity of ADAMTS18 cannot be distinctly illustrated in cells and mouse models with ADAMTS18 gene deletion. Thus, it could be speculated that pro-tumor functions or anti-oncogenic properties elicited by the ADAMTS18 may depend on the substrates or interacting partners present in the cell microenvironment.

## 7 ADAMTS18 as a drug target in cancer

Since ADAMTS18 has the potential to be a prognostic indicator for a variety of cancers, it is of great significance to study ADAMTS18 as an anticancer-targeted drug. So far, there is a gap in this research although ADAMTS18 in combination with some drugs has shown therapeutic effects in the field of anti-cancer (24–28). ADAMTS18 not only reverses the resistance of ccRCC to sunitinib and axitinib in combination with curcumin but also independently increases the sensitivity of lung cancer cells to cisplatin (26–28). Cisplatin is a widely used anticancer drug in the clinic, but its resistance reduces its clinical value (109, 110). Previous data showed that ADAMTS18 increases the sensitivity of lung cancer cells to cisplatin, thereby improving patient survival to some extent (28). As mentioned earlier, ADAMTS18 was found to inhibit the proliferation, migration, and invasion of lung cancer cells and to block lung cancer cells in the G0/G1 phase, suggesting that ADAMTS18 itself has a tumor-suppressing effect (28). ADAMTS18 has also been illustrated to increase lung cancer cell sensitivity to cisplatin by suppressing the EGFR/AKT signaling pathway (28). In addition, a new study revealed that the expression level of ADAMTS18 in HER2-positive breast tumor samples at the initial stage of post trastuzumab treatment is higher than that in recurrent HER2-positive tumor samples after post trastuzumab treatment, suggesting that ADAMTS18 can be responsible for a reference factor for drug resistance therapy (57).

Curcumin, as a medicinal plant, has become a hotspot of tumor research in recent years due to its excellent anti-inflammatory and antioxidant effects (111, 112). ADAMTS18 can assist curcumin in inhibiting the occurrence and development of ccRCC. Curcumin down-regulates the NF-κB and AKT signaling pathways, reverses the methylation of ADAMTS18 in ccRCC, and inhibits the growth of cancer cells by up-regulating ADAMTS18 expression (24). In addition, curcumin inhibits autophagy in cancer cells and achieves its tumor-suppressive effect by modulating the positive feedback mechanism between miR-148 and ADAMTS18 (25). Sunitinib and axitinib, as tyrosine kinase inhibitors with strong anti-tumor cell proliferation and anti-angiogenic effects, have been considered targeted drugs for the treatment of ccRCC, but their drug resistance is still an urgent clinical problem (113, 114). Curcumin was found to increase the sensitivity of sunitinib to ccRCC cells, and this mechanism is realized through the induction of ferroptosis by ADAMTS18 in ccRCC cells (26). The Chinese herbal compound SanHuang decoction containing curcumin can reverse the drug resistance of axitinib in ccRCC cells by up-regulating the expression of ADAMTS18 (27). In addition, the Chinese herbal compound SanHuang decoction and ADAMTS18 have a synergistic effect by increasing the proportion of CD8+ and CD4+T cells and reducing the ratio of CD45+/PD-L1 to increase tumor immune infiltration and inhibiting immune escape (27, 115).

## 8 Conclusions and future directions

ADAMTS18 fulfills multiple distinct roles in tumor tissues, affecting intracellular signals and tumor microenvironment, which is reflected in the complexity of biological functions and different expression patterns of ADAMTS18 in different types of tumor tissues (19, 20). From an oncological point of view, the relationship between the tumor-promoting and anti-tumorigenic properties of ADAMTS18 and specific substrates and interacting proteins has not been well explained, although the biological functions of ADAMTS18 have been extensively reported in the literature. In addition, the information provided by the established ADAMTS18 cell and mouse models is still unconvincing without the support of extensive clinical data. Therefore, more studies on the molecular mechanisms by which ADAMTS18 regulates cancer are needed in the future, which will not only contribute to the understanding of the physiological mechanisms of ADAMTS family members but also to the development of less toxic cancer therapies.

## Author contributions

WG: Writing – original draft. YZ: Conceptualization, Funding acquisition, Project administration, Supervision, Writing – original draft, Writing – review & editing.

## Glossary

**Table:**

ADAMTS	A disintegrin and metalloproteinase with thrombospondin motifs
TSR	Thrombospondin type motif
PLAC	Protease and lacunin
GEPIA	Gene expresssion profiling interactive analysis
CGI	CpG isands
TSG	Tumor suppressor gene
OS	Overall survival
DFS	Disease-free survival
IDC	Invasive ductal carcinoma
ER	Estrogen receptor
PR	Progesterone receptor
ESCC	Esophageal squamous cell carcinoma
HRGs	Hypoxia-related genes
CAC	Colitis-associated colon cancer
ccRCC	Clear cell renal cell carcinoma
TP53	Tumor suppressor protein p53
RCC	Renal cell carcinoma
EGFR	Epidermal growth factor receptor
EMT	Epithelial-mesenchymal transition
AKT	Protein kinase B
TME	Tumor microenvironment
ECM	Extracellular matrix
NK	Natural killer cell
DC	Dendritic cell
NKT2	Type 2 natural killer T cell
APC	Antigen-presenting cell
RBC	Red blood cell
Hb	Hemoglobin
AOAR	Aortic arch
CCA	Common carotid artery
CNCC	Cranial neural crest cells
ISV	Intersegment vessel
CPV	Caudal vein plexus
CV	Caudal vein
CCV	Common cardinal vein
GAG	Glycosaminoglycans
FAK	Focal adhesion kinase

## References

[1] Apte SS. A disintegrin-like and metalloprotease (reprolysin-type) with thrombospondin type 1 motif (ADAMTS) superfamily: functions and mechanisms. J Biol Chem. (2009) 284:31493–7. doi: 10.1074/jbc.R109.052340 PMC2797218 19734141

[2] Kelwick R Desanlis I Wheeler GN Edwards DR. The ADAMTS (A disintegrin and metalloproteinase with thrombospondin motifs) family. Genome Biol. (2015) 16:113. doi: 10.1186/s13059-015-0676-3 26025392 PMC4448532

[3] Kuno K Kanada N Nakashima E Fujiki F Ichimura F Matsushima K. Molecular cloning of a gene encoding a new type of metalloproteinase-disintegrin family protein with thrombospondin motifs as an inflammation associated gene. J Biol Chem. (1997) 272:556–62. doi: 10.1074/jbc.272.1.556 8995297

[4] Canals F Colomé N Ferrer C Plaza-Calonge Mdel C Rodríguez-Manzaneque JC. Identification of substrates of the extracellular protease ADAMTS1 by DIGE proteomic analysis. Proteomics. (2006) Suppl:S28–35. doi: 10.1002/pmic.200500446 16511810

[5] Wang WM Lee S Steiglitz BM Scott IC Lebares CC Allen ML. Transforming growth factor-beta induces secretion of activated ADAMTS-2. a procollagen III n-proteinase. J Biol Chem. (2003) 278:19549–57. doi: 10.1074/jbc.M300767200 12646579

[6] Matthews RT Gary SC Zerillo C Pratta M Solomon K Arner EC. Brain-enriched hyaluronan binding (BEHAB)/brevican cleavage in a glioma cell line is mediated by a disintegrin and metalloproteinase with thrombospondin motifs (ADAMTS) family member. J Biol Chem. (2000) 275:22695–703. doi: 10.1074/jbc.M909764199 10801887

[7] Nakada M Miyamori H Kita D Takahashi T Yamashita J Sato H. Human glioblastomas overexpress ADAMTS-5 that degrades brevican. Acta Neuropathol. (2005) 110:239–46. doi: 10.1007/s00401-005-1032-6 16133547

[8] Collins-Racie LA Flannery CR Zeng W Corcoran C Annis-Freeman B Agostino MJ. ADAMTS-8 exhibits aggrecanase activity and is expressed in human articular cartilage. Matrix Biol. (2004) 23:219–30. doi: 10.1016/j.matbio.2004.05.004 15296936

[9] Somerville RP Longpre JM Jungers KA Engle JM Ross M Evanko S. Characterization of ADAMTS-9 and ADAMTS-20 as a distinct ADAMTS subfamily related to caenorhabditis elegans GON-1. J Biol Chem. (2003) 278:9503–13. doi: 10.1074/jbc.M211009200 12514189

[10] Liu CJ Kong W Xu K Luan Y Ilalov K Sehgal B. ADAMTS-12 associates with and degrades cartilage oligomeric matrix protein. J Biol Chem. (2006) 281:15800–8. doi: 10.1074/jbc.M513433200 PMC1483932 16611630

[11] Fujikawa K Suzuki H McMullen B Chung D. Purification of human von willebrand factor-cleaving protease and its identification as a new member of the metalloproteinase family. Blood. (2001) 98:1662–6. doi: 10.1182/blood.v98.6.1662 11535495

[12] Dancevic CM Fraser FW Smith AD Stupka N Ward AC McCulloch DR. Biosynthesis and expression of a disintegrin-like and metalloproteinase domain with thrombospondin-1 repeats-15: a novel versican-cleaving proteoglycanase. J Biol Chem. (2013) 288:37267–76. doi: 10.1074/jbc.M112.418624 PMC3873579 24220035

[13] Gokce A Gokce EC Sunguroglu A. Role of adamts-1 in pleomorphic xanthoastrocytoma tumor cells progression. Turk Neurosurg. (2021) 31:731–9. doi: 10.5137/1019-5149.JTN.31011-20.3 34169995

[14] Binder MJ McCoombe S Williams ED McCulloch DR Ward AC. ADAMTS-15 has a tumor suppressor role in prostate cancer. Biomolecules. (2020) 10:682. doi: 10.3390/biom10050682 32354091 PMC7277637

[15] Fontanil T Rúa S Llamazares M Moncada-Pazos A Quirós PM García-Suárez O. Interaction between the ADAMTS-12 metalloprotease and fibulin-2 induces tumor-suppressive effects in breast cancer cells. Oncotarget. (2014) 5:1253–64. doi: 10.18632/oncotarget.1690 PMC4012729 24457941

[16] Martino-Echarri E Fernández-Rodríguez R Rodríguez-Baena FJ Barrientos-Durán A Torres-Collado AX Plaza-Calonge Mdel C. Contribution of ADAMTS1 as a tumor suppressor gene in human breast carcinoma. linking its tumor inhibitory properties to its proteolytic activity on nidogen-1 and nidogen-2. Int J Cancer. (2013) 133:2315–24. doi: 10.1002/ijc.28271 23681936

[17] Kelwick R Wagstaff L Decock J Roghi C Cooley LS Robinson SD. Metalloproteinase-dependent and -independent processes contribute to inhibition of breast cancer cell migration, angiogenesis and liver metastasis by a disintegrin and metalloproteinase with thrombospondin motifs-15. Int J Cancer. (2015) 136:E14–26. doi: 10.1002/ijc.29129 25099234

[18] Held-Feindt J Paredes EB Blömer U Seidenbecher C Stark AM Mehdorn HM. Matrix-degrading proteases ADAMTS4 and ADAMTS5 (disintegrins and metalloproteinases with thrombospondin motifs 4 and 5) are expressed in human glioblastomas. Int J Cancer. (2006) 118:55–61. doi: 10.1002/ijc.21258 16003758

[19] Jin H Wang X Ying J Wong AH Li H Lee KY. Epigenetic identification of ADAMTS18 as a novel 16q23.1 tumor suppressor frequently silenced in esophageal, nasopharyngeal and multiple other carcinomas. Oncogene. (2007) 26:7490–8. doi: 10.1038/sj.onc.1210559 PMC2875853 17546048

[20] Wei X Prickett TD Viloria CG Molinolo A Lin JC Cardenas-Navia I. NISC comparative sequencing program; rosenberg SA, davies MA, gershenwald JE, lópez-otín c, samuels y. mutational and functional analysis reveals ADAMTS18 metalloproteinase as a novel driver in melanoma. Mol Cancer Res. (2010) 8:1513–25. doi: 10.1158/1541-7786.MCR-10-0262 PMC3058631 21047771

[21] Jiang K Li L Xie Y Xie D Xiao Q. High ADAMTS18 expression is associated with poor prognosis in stomach adenocarcinoma. Oncol Lett. (2020) 20:211. doi: 10.3892/ol.2020.12074 32963617 PMC7491029

[22] Ataca D Aouad P Constantin C Laszlo C Beleut M Shamseddin M. The secreted protease Adamts18 links hormone action to activation of the mammary stem cell niche. Nat Commun. (2020) 11:1571. doi: 10.1038/s41467-020-15357-y 32218432 PMC7099066

[23] Lu T Lin X Pan YH Yang N Ye S Zhang Q. ADAMTS18 deficiency leads to pulmonary hypoplasia and bronchial microfibril accumulation. iScience. (2020) 23:101472. doi: 10.1016/j.isci.2020.101472 32882513 PMC7476315

[24] Xu B Peng YJ Zhu WJ. Curcumin inhibits viability of clear cell renal cell carcinoma by down-regulating ADAMTS18 gene methylation though NF-κ b and AKT signaling pathway. Chin J Integr Med. (2022) 28:419–24. doi: 10.1007/s11655-021-3445-z 33997938

[25] Xu B Yuan CW Zhang JE. Curcumin inhibits proliferation of renal cell carcinoma *in vitro* and *in vivo* by regulating miR-148/ADAMTS18 through suppressing autophagy. Chin J Integr Med. (2023) 29:699–706. doi: 10.1007/s11655-022-3690-9 36477451

[26] Xu B Zhu WJ Peng YJ Cheng SD. Curcumin reverses the sunitinib resistance in clear cell renal cell carcinoma (ccRCC) through the induction of ferroptosis *via* the ADAMTS18 gene. Transl Cancer Res. (2021) 10:3158–67. doi: 10.21037/tcr-21-227 PMC8797884 35116623

[27] Xu B Zhang J Ye L Yuan C. Chinese herbal compound SanHuang decoction reverses axitinib resistance in ccRCC through regulating immune cell infiltration by affecting ADAMTS18 expression. Am J Cancer Res. (2023) 13:2841–60. PMC10408491 37560000

[28] Zhang Y Xu H Mu J Guo S Ye L Li D. Inactivation of ADAMTS18 by aberrant promoter hypermethylation contribute to lung cancer progression. J Cell Physiol. (2019) 234:6965–75. doi: 10.1002/jcp.27439 30417422

[29] Théret N Bouezzedine F Azar F Diab-Assaf M Legagneux V. ADAM and ADAMTS proteins, new players in the regulation of hepatocellular carcinoma microenvironment. Cancers (Basel). (2021) 13:1563. doi: 10.3390/cancers13071563 33805340 PMC8037375

[30] Foulcer SJ Nelson CM Quintero MV Kuberan B Larkin J Dours-Zimmermann MT. Determinants of versican-V1 proteoglycan processing by the metalloproteinase ADAMTS5. J Biol Chem. (2014) 289:27859–73. doi: 10.1074/jbc.M114.573287 PMC4183820 25122765

[31] Gao W Zhu J Westfield LA Tuley EA Anderson PJ Sadler JE. Rearranging exosites in noncatalytic domains can redirect the substrate specificity of ADAMTS proteases. J Biol Chem. (2012) 287:26944–52. doi: 10.1074/jbc.M112.380535 PMC3411030 22707719

[32] Kashiwagi M Enghild JJ Gendron C Hughes C Caterson B Itoh Y. Altered proteolytic activities of ADAMTS-4 expressed by c-terminal processing. J Biol Chem. (2004) 279:10109–19. doi: 10.1074/jbc.M312123200 14662755

[33] Gao W Anderson PJ Majerus EM Tuley EA Sadler JE. Exosite interactions contribute to tension-induced cleavage of von willebrand factor by the antithrombotic ADAMTS13 metalloprotease. Proc Natl Acad Sci USA. (2006) 103:19099–104. doi: 10.1073/pnas.0607264104 PMC1681350 17146059

[34] Mougin Z Huguet Herrero J Boileau C Le Goff C. ADAMTS proteins and vascular remodeling in aortic aneurysms. Biomolecules. (2021) 12:12. doi: 10.3390/biom12010012 35053160 PMC8773774

[35] Kumar S Rao N Ge R. Emerging roles of ADAMTSs in angiogenesis and cancer. Cancers (Basel). (2012) 4:1252–99. doi: 10.3390/cancers4041252 PMC3712723 24213506

[36] Li Z Nardi MA Li YS Zhang W Pan R Dang S. C-terminal ADAMTS-18 fragment induces oxidative platelet fragmentation, dissolves platelet aggregates, and protects against carotid artery occlusion and cerebral stroke. Blood. (2009) 113:6051–60. doi: 10.1182/blood-2008-07-170571 PMC2699219 19218546

[37] Mosyak L Georgiadis K Shane T Svenson K Hebert T McDonagh T. Crystal structures of the two major aggrecan degrading enzymes, ADAMTS4 and ADAMTS5. Protein Sci. (2008) 17:16–21. doi: 10.1110/ps.073287008 18042673 PMC2144589

[38] Shieh HS Mathis KJ Williams JM Hills RL Wiese JF Benson TE. High resolution crystal structure of the catalytic domain of ADAMTS-5 (aggrecanase-2). J Biol Chem. (2008) 283:1501–7. doi: 10.1074/jbc.M705879200 17991750

[39] Nordgard SH Johansen FE Alnaes GI Bucher E Syvänen AC Naume B. Genome-wide analysis identifies 16q deletion associated with survival, molecular subtypes, mRNA expression, and germline haplotypes in breast cancer patients. Genes Chromosomes Cancer. (2008) 47:680–96. doi: 10.1002/gcc.20569 18398821

[40] Li Z Zhang W Shao Y Zhang C Wu Q Yang H. High-resolution melting analysis of ADAMTS18 methylation levels in gastric, colorectal and pancreatic cancers. Med Oncol. (2010) 27:998–1004. doi: 10.1007/s12032-009-9323-8 19806480

[41] Cao W Lee H Wu W Zaman A McCorkle S Yan M. Multi-faceted epigenetic dysregulation of gene expression promotes esophageal squamous cell carcinoma. Nat Commun. (2020) 11:3675. doi: 10.1038/s41467-020-17227-z 32699215 PMC7376194

[42] Lu T Dang S Zhu R Wang Y Nie Z Hong T. Adamts18 deficiency promotes colon carcinogenesis by enhancing β-catenin and p38MAPK/ERK1/2 signaling in the mouse model of AOM/DSS-induced colitis-associated colorectal cancer. Oncotarget. (2017) 8:18979–90. doi: 10.18632/oncotarget.14866 PMC5386663 28145888

[43] Xu H Xiao Q Fan Y Xiang T Li C Li C. Epigenetic silencing of ADAMTS18 promotes cell migration and invasion of breast cancer through AKT and NF-κB signaling. Cancer Med. (2017) 6:1399–408. doi: 10.1002/cam4.1076 PMC5463072 28503860

[44] Zhang L Liu Y Zheng P. Downregulation of ADAMTS18 may serve as a poor prognostic biomarker for cervical cancer patients. Appl Immunohistochem Mol Morphol. (2018) 26:670–5. doi: 10.1097/PAI.0000000000000496 28362704

[45] Xu B Zhang L Luo C Qi Y Cui Y Ying JM. Hypermethylation of the 16q23.1 tumor suppressor gene ADAMTS18 in clear cell renal cell carcinoma. Int J Mol Sci. (2015) 16:1051–65. doi: 10.3390/ijms16011051 PMC4307290 25569086

[46] Sjöblom T Jones S Wood LD Parsons DW Lin J Barber TD. The consensus coding sequences of human breast and colorectal cancers. Science. (2006) 314:268–74. doi: 10.1126/science.1133427 16959974

[47] Wood LD Parsons DW Jones S Lin J Sjöblom T Leary RJ. The genomic landscapes of human breast and colorectal cancers. Science. (2007) 318:1108–13. doi: 10.1126/science.1145720 17932254

[48] Kılıç MÖ Aynekin B Bozer M Kara A Haltaş H İçen D. Differentially regulated ADAMTS1, 8, 9, and 18 in pancreas adenocarcinoma. Prz Gastroenterol. (2017) 12:262–70. doi: 10.5114/pg.2017.72101 PMC5771450 29358995

[49] Dai X Ren T Zhang Y Nan N. Methylation multiplicity and its clinical values in cancer. Expert Rev Mol Med. (2021) 23:e2. doi: 10.1017/erm.2021.4 33787478 PMC8086398

[50] Han YD Oh TJ Chung TH Jang HW Kim YN An S. Early detection of colorectal cancer based on presence of methylated syndecan-2 (SDC2) in stool DNA. Clin Epigenetics. (2019) 11:51. doi: 10.1186/s13148-019-0642-0 30876480 PMC6419806

[51] Xu RH Wei W Krawczyk M Wang W Luo H Flagg K. Circulating tumour DNA methylation markers for diagnosis and prognosis of hepatocellular carcinoma. Nat Mater. (2017) 16:1155–61. doi: 10.1038/nmat4997 29035356

[52] Papanicolau-Sengos A Aldape K. DNA methylation profiling: An emerging paradigm for cancer diagnosis. Annu Rev Pathol. (2022) 17:295–321. doi: 10.1146/annurev-pathol-042220-022304 34736341

[53] Kilic MO Aynekin B Kara A Icen D Demircan K. Differentially regulated ADAMTS1, 8, and 18 in gastric adenocarcinoma. Bratisl Lek Listy. (2017) 118:71–6. doi: 10.4149/BLL_2017_014 28814085

[54] Guo X Li J Zhang H Liu H Liu Z Wei X. Relationship between ADAMTS8, ADAMTS18, and ADAMTS20 (A disintegrin and metalloproteinase with thrombospondin motifs) expressions and tumor molecular classification, clinical pathological parameters, and prognosis in breast invasive ductal carcinoma. Med Sci Monit. (2018) 24:3726–35. doi: 10.12659/MSM.907310 PMC6014152 29860265

[55] Yip CH Rhodes A. Estrogen and progesterone receptors in breast cancer. Future Oncol. (2014) 10:2293–301. doi: 10.2217/fon.14.110 25471040

[56] Xiao W Tang P Sui Z Han Y Zhao G Wu X. Establishment of a risk model by integrating hypoxia genes in predicting prognosis of esophageal squamous cell carcinoma. Cancer Med. (2023) 12:2117–33. doi: 10.1002/cam4.5002 PMC9883439 35789548

[57] Nie J Dang S Zhu R Lu T Zhang W. ADAMTS18 deficiency associates extracellular matrix dysfunction with a higher risk of HER2-positive mammary tumorigenesis and metastasis. Breast Cancer Res. (2024) 26:19. doi: 10.1186/s13058-024-01771-3 38287441 PMC10826190

[58] Jiang L Kon N Li T Wang SJ Su T Hibshoosh H. Ferroptosis as a p53-mediated activity during tumour suppression. Nature. (2015) 520:57–62. doi: 10.1038/nature14344 25799988 PMC4455927

[59] Reichert CO de Freitas FA Sampaio-Silva J Rokita-Rosa L Barros PL Levy D. Ferroptosis mechanisms involved in neurodegenerative diseases. Int J Mol Sci. (2020) 21:8765. doi: 10.3390/ijms21228765 33233496 PMC7699575

[60] Xiu Z Zhu Y Han J Li Y Yang X Yang G. Caryophyllene oxide induces ferritinophagy by regulating the NCOA4/FTH1/LC3 pathway in hepatocellular carcinoma. Front Pharmacol. (2022) 13:930958. doi: 10.3389/fphar.2022.930958 35899120 PMC9313605

[61] Jones TM Carew JS Nawrocki ST. Therapeutic targeting of autophagy for renal cell carcinoma therapy. Cancers (Basel). (2020) 12:1185. doi: 10.3390/cancers12051185 32392870 PMC7281213

[62] Julien S Puig I Caretti E Bonaventure J Nelles L van Roy F. Activation of NF-kappaB by akt upregulates snail expression and induces epithelium mesenchyme transition. Oncogene. (2007) 26:7445–56. doi: 10.1038/sj.onc.1210546 17563753

[63] Nusse R Clevers H. Wnt/β-catenin signaling, disease, and emerging therapeutic modalities. Cell. (2017) 169:985–99. doi: 10.1016/j.cell.2017.05.016 28575679

[64] Vermeulen L De Sousa E Melo F van der Heijden M Cameron K de Jong JH Borovski T. Wnt activity defines colon cancer stem cells and is regulated by the microenvironment. Nat Cell Biol. (2010) 12:468–76. doi: 10.1038/ncb2048 20418870

[65] Hao Y Baker D Ten Dijke P. TGF-β-Mediated epithelial-mesenchymal transition and cancer metastasis. Int J Mol Sci. (2019) 20:2767. doi: 10.3390/ijms20112767 31195692 PMC6600375

[66] Na TY Schecterson L Mendonsa AM Gumbiner BM. The functional activity of e-cadherin controls tumor cell metastasis at multiple steps. Proc Natl Acad Sci USA. (2020) 117:5931–7. doi: 10.1073/pnas.1918167117 PMC7084067 32127478

[67] Xiao S Liu L Lu X Long J Zhou X Fang M. The prognostic significance of bromodomain PHD-finger transcription factor in colorectal carcinoma and association with vimentin and e-cadherin. J Cancer Res Clin Oncol. (2015) 141:1465–74. doi: 10.1007/s00432-015-1937-y PMC11823721 25716692

[68] Choi GC Li J Wang Y Li L Zhong L Ma B. The metalloprotease ADAMTS8 displays antitumor properties through antagonizing EGFR-MEK-ERK signaling and is silenced in carcinomas by CpG methylation. Mol Cancer Res. (2014) 12:228–38. doi: 10.1158/1541-7786 24184540

[69] Llamazares M Obaya AJ Moncada-Pazos A Heljasvaara R Espada J López-Otín C. The ADAMTS12 metalloproteinase exhibits anti-tumorigenic properties through modulation of the ras-dependent ERK signalling pathway. J Cell Sci. (2007) 120:3544–52. doi: 10.1242/jcs.005751 17895370

[70] Paillas S Boissière F Bibeau F Denouel A Mollevi C Causse A. Targeting the p38 MAPK pathway inhibits irinotecan resistance in colon adenocarcinoma. Cancer Res. (2011) 71:1041–9. 10.1158/0008-5472.CAN-10-2726 PMC3304472 21159664

[71] Comes F Matrone A Lastella P Nico B Susca FC Bagnulo R. A novel cell type-specific role of p38alpha in the control of autophagy and cell death in colorectal cancer cells. Cell Death Differ. (2007) 14:693–702. doi: 10.1038/sj.cdd.4402076 17159917

[72] Belli C Trapani D Viale G D'Amico P Duso BA Della Vigna P. Targeting the microenvironment in solid tumors. Cancer Treat Rev. (2018) 65:22–32. doi: 10.1016/j.ctrv.2018.02.004 29502037

[73] Hinshaw DC Shevde LA. The tumor microenvironment innately modulates cancer progression. Cancer Res. (2019) 79(18):4557–66. doi: 10.1158/0008-5472.CAN-18-3962 PMC6744958 31350295

[74] Xiao Y Yu D. Tumor microenvironment as a therapeutic target in cancer. Pharmacol Ther. (2021) 221:107753. doi: 10.1016/j.pharmthera.2020.107753 33259885 PMC8084948

[75] Cal S López-Otín C. ADAMTS proteases and cancer. Matrix Biol. (2015) 44 -46:77–85. doi: 10.1016/j.matbio.2015.01.013 25636539

[76] Greten FR Grivennikov SI. Inflammation and cancer: Triggers, mechanisms, and consequences. Immunity. (2019) 51:27–41. doi: 10.1016/j.immuni.2019.06.025 31315034 PMC6831096

[77] Hanahan D Weinberg RA. Hallmarks of cancer: the next generation. Cell. (2011) 144:646–74. doi: 10.1016/j.cell.2011.02.013 21376230

[78] Denk D Greten FR. Inflammation: the incubator of the tumor microenvironment. Trends Cancer. (2022) 8:901–14. doi: 10.1016/j.trecan.2022.07.002 35907753

[79] Zelenay S van der Veen AG Böttcher JP Snelgrove KJ Rogers N Acton SE. Cyclooxygenase-dependent tumor growth through evasion of immunity. Cell. (2015) 162:1257–70. doi: 10.1016/j.cell.2015.08.015 PMC4597191 26343581

[80] Grivennikov S Karin E Terzic J Mucida D Yu GY Vallabhapurapu S. IL-6 and Stat3 are required for survival of intestinal epithelial cells and development of colitis-associated cancer. Cancer Cell. (2009) 15:103–13. doi: 10.1016/j.ccr.2009.01.001 PMC2667107 19185845

[81] Redondo-García S Peris-Torres C Caracuel-Peramos R Rodríguez-Manzaneque JC. ADAMTS proteases and the tumor immune microenvironment: Lessons from substrates and pathologies. Matrix Biol Plus. (2020) 9:100054. doi: 10.1016/j.mbplus.2020.100054 33718860 PMC7930849

[82] Locy H de Mey S de Mey W De Ridder M Thielemans K Maenhout SK. Immunomodulation of the tumor microenvironment: Turn foe into friend. Front Immunol. (2018) 9:2909. doi: 10.3389/fimmu.2018.02909 30619273 PMC6297829

[83] Xie W Medeiros LJ Li S Yin CC Khoury JD Xu J. PD-1/PD-L1 pathway and its blockade in patients with classic hodgkin lymphoma and non-hodgkin large-cell lymphomas. Curr Hematol Malig Rep. (2020) 15:372–81. doi: 10.1007/s11899-020-00589-y 32394185

[84] Lv B Wang Y Ma D Cheng W Liu J Yong T. Immunotherapy: Reshape the tumor immune microenvironment. Front Immunol. (2022) 13:844142. doi: 10.3389/fimmu.2022.844142 35874717 PMC9299092

[85] You W Ouyang J Cai Z Chen Y Wu X. Comprehensive analyses of immune subtypes of stomach adenocarcinoma for mRNA vaccination. Front Immunol. (2022) 13:827506. doi: 10.3389/fimmu.2022.827506 35874675 PMC9300892

[86] Polak P Fu L Foulkes WD. PD-1 and PD-L1 blockade plus chemotherapy in endometrial cancer. N Engl J Med. (2023) 389:866. doi: 10.1056/NEJMc2308037 37646693

[87] Huang C Ren S Chen Y Liu A Wu Q Jiang T. PD-L1 methylation restricts PD-L1/PD-1 interactions to control cancer immune surveillance. Sci Adv. (2023) 9:eade4186. doi: 10.1126/sciadv.ade4186 37235656 PMC10219601

[88] D'Alterio C Buoncervello M Ieranò C Napolitano M Portella L Rea G. Targeting CXCR4 potentiates anti-PD-1 efficacy modifying the tumor microenvironment and inhibiting neoplastic PD-1. J Exp Clin Cancer Res. (2019) 38:432. doi: 10.1186/s13046-019-1420-8 31661001 PMC6819555

[89] Bhat SM Badiger VA Vasishta S Chakraborty J Prasad S Ghosh S. 3D tumor angiogenesis models: recent advances and challenges. J Cancer Res Clin Oncol. (2021) 147:3477–94. doi: 10.1007/s00432-021-03814-0 PMC8557138 34613483

[90] Roda N Blandano G Pelicci PG. Blood vessels and peripheral nerves as key players in cancer progression and therapy resistance. Cancers (Basel). (2021) 13:4471. doi: 10.3390/cancers13174471 34503281 PMC8431382

[91] Dang S Bu D Lu T Wang Z Liu J Zhang W. Adamts18 deficiency increases arterial thrombus formation associated with vascular defects in mice. Biochem Biophys Res Commun. (2018) 496:1362–8. doi: 10.1016/j.bbrc.2018.02.032 29421655

[92] Lu T Zhang T Wang C Yang N Pan YH Dang S. Adamts18 deficiency in zebrafish embryo causes defective trunk angiogenesis and caudal vein plexus formation. Biochem Biophys Res Commun. (2020) 521:907–13. doi: 10.1016/j.bbrc.2019.10.202 31711643

[93] Ye S Yang N Lu T Wu T Wang L Pan YH. Adamts18 modulates the development of the aortic arch and common carotid artery. iScience. (2021) 24:102672. doi: 10.1016/j.isci.2021.102672 34189436 PMC8215225

[94] Majidpoor J Mortezaee K. Angiogenesis as a hallmark of solid tumors – clinical perspectives. Cell Oncol (Dordr). (2021) 44:715–37. doi: 10.1007/s13402-021-00602-3 PMC12980750 33835425

[95] Mushimiyimana I Niskanen H Beter M Laakkonen JP Kaikkonen MU Ylä-Herttuala S. Characterization of a functional endothelial super-enhancer that regulates ADAMTS18 and angiogenesis. Nucleic Acids Res. (2021) 49:8078–96. doi: 10.1093/nar/gkab633 PMC8373076 34320216

[96] Kim MS Ha SE Wu M Zogg H Ronkon CF Lee MY. Extracellular matrix biomarkers in colorectal cancer. Int J Mol Sci. (2021) 22:9185. doi: 10.3390/ijms22179185 34502094 PMC8430714

[97] Bonnans C Chou J Werb Z. Remodelling the extracellular matrix in development and disease. Nat Rev Mol Cell Biol. (2014) 15:786–801. doi: 10.1038/nrm3904 25415508 PMC4316204

[98] Jiang Y Zhang H Wang J Liu Y Luo T Hua H. Targeting extracellular matrix stiffness and mechanotransducers to improve cancer therapy. J Hematol Oncol. (2022) 15:34. doi: 10.1186/s13045-022-01252-0 35331296 PMC8943941

[99] Dong Y Zheng Q Wang Z Lin X You Y Wu S. Higher matrix stiffness as an independent initiator triggers epithelial-mesenchymal transition and facilitates HCC metastasis. J Hematol Oncol. (2019) 12:112. doi: 10.1186/s13045-019-0795-5 31703598 PMC6839087

[100] Humphrey JD Dufresne ER Schwartz MA. Mechanotransduction and extracellular matrix homeostasis. Nat Rev Mol Cell Biol. (2014) 15:802–12. doi: 10.1038/nrm3896 PMC4513363 25355505

[101] Dalton CJ Lemmon CA. Fibronectin: Molecular structure, fibrillar structure and mechanochemical signaling. Cells. (2021) 10:2443. doi: 10.3390/cells10092443 34572092 PMC8471655

[102] Kadler KE Hill A Canty-Laird EG. Collagen fibrillogenesis: fibronectin, integrins, and minor collagens as organizers and nucleators. Curr Opin Cell Biol. (2008) 20:495–501. doi: 10.1016/j.ceb.2008.06.008 18640274 PMC2577133

[103] Filla MS Dimeo KD Tong T Peters DM. Disruption of fibronectin matrix affects type IV collagen, fibrillin and laminin deposition into extracellular matrix of human trabecular meshwork (HTM) cells. Exp Eye Res. (2017) 165:7–19. doi: 10.1016/j.exer.2017.08.017 28860021 PMC5705399

[104] Park J Schwarzbauer JE. Mammary epithelial cell interactions with fibronectin stimulate epithelial-mesenchymal transition. Oncogene. (2014) 33:1649–57. doi: 10.1038/onc.2013.118 PMC3934944 23624917

[105] Zhang X Teng X Liu HH. The expression of fibronectin in breast cancer and the relationship between FN and lymphanoid metastasis. Henan J Oncol. (2000) 02:79–80.

[106] Cagnet S Faraldo MM Kreft M Sonnenberg A Raymond K Glukhova MA. Signaling events mediated by α3β1 integrin are essential for mammary tumorigenesis. Oncogene. (2014) 33:4286–95. doi: 10.1038/onc.2013.391 24077284

[107] Fu Y Zhang Y Lei Z Liu T Cai T Wang A. Abnormally activated OPN/integrin αVβ3/FAK signalling is responsible for EGFR-TKI resistance in EGFR mutant non-small-cell lung cancer. J Hematol Oncol. (2020) 13:169. doi: 10.1186/s13045-020-01009-7 33287873 PMC7720454

[108] Rubashkin MG Cassereau L Bainer R DuFort CC Yui Y Ou G. Force engages vinculin and promotes tumor progression by enhancing PI3K activation of phosphatidylinositol (3,4,5)-triphosphate. Cancer Res. (2014) 74:4597–611. doi: 10.1158/0008-5472 PMC4191931 25183785

[109] Dasari S Tchounwou PB. Cisplatin in cancer therapy: molecular mechanisms of action. Eur J Pharmacol. (2014) 740:364–78. doi: 10.1016/j.ejphar.2014.07.025 PMC4146684 25058905

[110] Ghosh S. Cisplatin: The first metal based anticancer drug. Bioorg Chem. (2019) 88:102925. doi: 10.1016/j.bioorg.2019.102925 31003078

[111] Tomeh MA Hadianamrei R Zhao X. A review of curcumin and its derivatives as anticancer agents. Int J Mol Sci. (2019) 20:1033. doi: 10.3390/ijms20051033 30818786 PMC6429287

[112] Sadeghi M Dehnavi S Asadirad A Xu S Majeed M Jamialahmadi T. Curcumin and chemokines: mechanism of action and therapeutic potential in inflammatory diseases. Inflammopharmacology. (2023) 31:1069–93. doi: 10.1007/s10787-023-01136-w PMC10062691 36997729

[113] Gupta R Maitland ML. Sunitinib, hypertension, and heart failure: a model for kinase inhibitor-mediated cardiotoxicity. Curr Hypertens Rep. (2011) 13:430–5. doi: 10.1007/s11906-011-0229-4 21931979

[114] Riesenbeck LM Bierer S Hoffmeister I Köpke T Papavassilis P Hertle L. Hypothyroidism correlates with a better prognosis in metastatic renal cancer patients treated with sorafenib or sunitinib. World J Urol. (2011) 29:807–13. doi: 10.1007/s00345-010-0627-2 21153827

[115] Hou Y Liang HL Yu X Liu Z Cao X Rao E. Radiotherapy and immunotherapy converge on elimination of tumor-promoting erythroid progenitor cells through adaptive immunity. Sci Transl Med. (2021) 13:eabb0130. doi: 10.1126/scitranslmed.abb0130 33627484 PMC8710940

